# Assessing Climate Benefits and Circularity of Using Glass Waste in Concrete and New Glass Production

**DOI:** 10.3390/ma19091750

**Published:** 2026-04-24

**Authors:** Madumita Sadagopan, Abdinasir Kadawo, Habib Loubani, Nada Al-Hellali, Nitin Harale, Agnes Nagy

**Affiliations:** Resource Recovery and Building Technology, University of Borås, 50 190 Borås, Sweden; madumita.sadagopan@hb.se (M.S.); abdinasir.kadawo@hb.se (A.K.); habib.loubani@hb.se (H.L.); nitin.harale@hb.se (N.H.); agnes.nagy@hb.se (A.N.)

**Keywords:** SCM, circularity, climate reduction, concrete, glass, open-loop recycling, closed-loop recycling

## Abstract

**Highlights:**

- The avoided CO_2_ burden for recycling glass waste to new glass is 0.945 ton CO_2_eq/ton glass waste compared to glass waste as cement replacement with 0.776 ton CO_2_eq/ton glass waste.
- Only 3% postconsumer cullet or demolition glass is usable in the production of new flat glass.
- Up to 20% cement and 13% sand can be replaced by demolition glass in concrete without compromising compressive strength.
- Closed-loop recycling results in higher CO_2_ savings but accommodates smaller volumes of demolition glass waste.
- Glass waste is used to its highest potential through closed-loop recycling.
- By exercising both recycling options together, glass producers can access larger volumes of demolition glass waste, thus maximizing CO_2_ savings and circularity.

**Abstract:**

Flat glass waste from building demolition is an underused resource with potential to reduce the climate impact of construction materials. This study compares two recycling pathways for flat glass waste: the first is closed-loop recycling into new glass, and the second is the use of glass in concrete as a replacement for cement. The comparison is based on life cycle, circularity assessment and experimental evaluation of concrete performance. Recycling flat glass into new glass can reduce emissions by 945 kg CO_2_eq per ton of recycled glass when the production mix contains 65 percent recycled content. However, only between 1 and 3% percent of demolition flat glass is suitable for this process because of contamination and quality limitations. As a result, the practical climate benefit of demolition glass in new glass production is limited to about 38 kg CO_2_eq per ton of demolition glass. Concrete offers a much larger waste sink. Replacing 20% of cement with milled glass powder results in emission savings of 776 kg CO_2_eq per ton of glass. A concrete mix containing 33% glass shows the same compressive strength as a reference mix.

## 1. Introduction

The construction sector has been identified as a focus area within the EU’s transition to a circular economy. Construction-related activities consume 50% of all extracted materials and generate about 35% of the total waste in the EU [1]. Management of waste through reuse and recycling has been identified to significantly reduce climate impact through reduced greenhouse gas emissions in addition to reduced extraction of virgin materials [2]. Recycling may not always be a feasible strategy for every waste type, especially from the perspective of climate impact reduction and reduction in virgin material extraction [3]. Therefore, waste management strategies should be supported by validation tools to assess climate impact reduction and circularity gains.

The focus of this article is a comparison of recycling alternatives for flat glass waste from building demolition, especially renovation of windows. The recycling alternatives are glass waste as a raw material in new glass production and glass waste as a cement replacement in concrete. The basis of the comparison is the climate impact reduction and circularity arising consequently to recycling glass waste by either alternative.

Flat glass waste is a major material flow from demolition, amounting to a yearly 1.5 million tons in Europe, arising mostly from the renovation of windows [4]. A large share of the glass waste is mismanaged, with 10,000–20,000 tons deposited in landfills or used as filling material in landfill construction [5]. The waste sorting operations ongoing at demolition sites ensure the availability of clean glass fractions fit for recycling to new glass and other building materials such as foam glass and glass wool [6].

### 1.1. Background

A previous study from the domain of glass recycling evaluates closed-loop recycling of flat glass waste, i.e., recycling glass waste into new glass based on an economic-environmental analysis [7]. The results of a Life Cycle Assessment (LCA) show 51% reduction in standardized total environmental impact; economic benefits may be realized with improvements in crushing and separation processes.

The assessment of circularity for closed-loop recycling of glass from previous research shows an indicator, the closed-loop recycling rate, measured by the amount of waste glass cullet used to produce new container glass [8]. There are many research articles in the literature discussing the circularity of glass recycling on a conceptual level; however, the circularity metrics are indirectly addressed by collection and recycling rates.

There is ample research conducted on milled glass as a cement replacement with a major focus on the technical aspects. This includes chemical and physical properties of the glass powder and the resulting mechanical properties and durability of concrete with glass powder as a cement replacement. A review article addressing the technical aspects with evidence from research literature has earlier been published by the authors of this article [9]. Among the many articles published on the environmental impacts of using glass powder in concrete, a Canadian study has conducted an LCA to validate the environmental feasibility of using glass as a cement replacement in concrete [10]. The LCA is based on a concrete prototype element, covering production, construction, use and end-of-life phases of a concrete sidewalk. The global warming potential for the concrete mix with 25% glass replacement is 20% less than the conventional mix composed solely of cement. The study does not address other competing recycling alternatives for glass waste such as closed-loop recycling, a superior alternative with regard to circular resource use. As the glass powder is produced from mixed glass waste sourced from landfills, it is understood that the glass waste is not suitable for closed-loop recycling. The study solely addresses the environmental benefits and does not address the increase in circularity of the concrete by the inclusion of waste glass compared to the reference concrete.

There is ample literature on the separate topics of closed-loop recycling of glass and recycling glass as a cement replacement. However, there is a lack of research in comparing which is the most feasible recycling alternative between the two for flat glass waste. Such comparison studies have been conducted for other types of waste such as construction and demolition waste, for example, comparison of incineration, landfilling and recycling of construction waste using a multi-criteria assessment model for economic and environmental assessment [11].

In general, recycling scenarios are evaluated for their environmental impacts using LCA-based tools and multi-criteria analysis. The circularity resulting from recycling is captured in different studies using LCA [7] and circularity indicators [8]. A combined evaluation of recycling alternatives based on environmental impact and circularity is missing in the literature, which this study tries to address.

#### 1.1.1. Closed-Loop Recycling: Glass Waste for New Glass Production

Glass waste or cullet is one of the raw materials in flat glass production besides sand, soda ash and limestone. An amount of 1 ton of cullet can replace 1.2 tons of raw material in glass production [12,13]. This results in a significant reduction in CO_2_ emissions up to 50% along with reduced extraction of virgin sand and raw materials.

**Figure 1:**
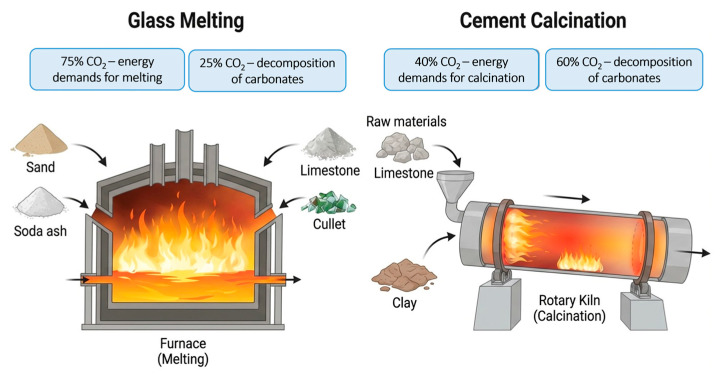
Raw materials and CO_2_ emissions arising from glass and cement manufacturing processes.

The CO_2_ emissions from glass production are substantial: 75% of CO_2_ emissions arise from energy demands to reach high melting temperatures. The rest 25% are released due to the breakdown of carbonates present in soda and limestone during melting [12]; the details are shown in Figure 1. With crushed glass as raw material it is possible to lower the melting temperature, which results in lowered energy demands by 2.5–3% with every 10% cullet replacement. The release of carbonates is partially reduced as glass waste has already undergone carbonate decomposition. This results in a total of 0.3 tons of CO_2_ savings with every 1 ton of cullet used in production [14].

Over 95% of the total cullet share is sourced from cutouts from glass production, also called preconsumer cullet. Preconsumer cullet is most suitable for closed-loop recycling due to its uniform quality [12,13]. The rising demands for preconsumer cullet have led to glass producers acquiring glass waste from demolition, an underutilized material flow [14]. The share of glass waste from demolition or postconsumer cullet is very low in glass production, between 1 and 3% of the total cullet content. This is due to the strict purity requirements on cullet, high processing costs and practical challenges such as the availability of suitable postconsumer cullet [14].

**Figure 2:**
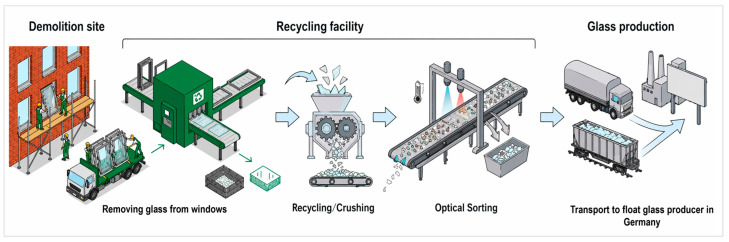
Recycling activities involved in closed-loop recycling of glass: a case study of Sweden.

The quality of glass waste from demolition is ensured by selective demolition of windows, recycling processes involving window frame–glass separation, cullet crushing and optical sorting to ensure purity of postconsumer glass cullet [15], as shown in Figure 2.

#### 1.1.2. Open-Loop Recycling: Glass Waste as Cement Replacement

Cement production is the main contributor to the CO_2_ footprint of concrete, accounting for 90% of concrete-related emissions [16], mainly due to the process and fuel-related emissions from the calcination process represented in Figure 1. The concrete industry is rapidly transitioning towards circularity and climate reduction, thus maximizing the use of supplementary cementitious materials (SCMs) in concrete. This has resulted in increasing demands for the use of industrial byproducts such as fly ash and blast furnace slag [17]. The CO_2_ emissions for byproducts are significantly low, as a major share of emissions is allocated towards the main product. For example, byproduct slag is allocated only 40 kg CO_2_eq/ton compared to steel, which is the main product with 1700 kg CO_2_eq/ton [9].

SCMs derived from waste flows are being investigated as potential cement replacements, for example, biochar derived from agricultural residues through pyrolysis. The partial replacement of cement with biochar brings reductions in CO_2_ emissions corresponding to the reduced cement content. Further CO_2_ reduction is possible when the stored biogenic carbon in the biochar is considered over the concrete’s service life [18].

Slag and biochar are also usable as aggregates in concrete and contribute to reducing the environmental impacts associated with the extraction of natural sand and crushed stone. Constituting the largest volume share in concrete, aggregate substitution offers notable circularity benefits compared to cement replacement. However, as cement has a larger carbon footprint, cement replacement brings larger climate benefits than aggregate replacement [3].

When glass is milled as fine as cement it reacts with byproducts of cement hydration, portlandite Ca(OH)_2_ to form cement gel [19]. With a particle grading where at least 95% of its particles are finer than 45-micron, and silica content larger than 60% glass powder meets the pozzolanic criteria in ASTM standard C1866M-20 [20], oxide content shown in Table 1. Glass powder as a cement replacement in concrete brings forth compressive strength likening concrete with 100% Portland cement for replacements 20–30% of the cement by weight [19].

A review study on glass as cement replacement has been conducted by the authors of this article; the results show that the best compressive strength results are achieved at cement replacements between 20 and 30%. Due to its pozzolanic behavior the glass contributes to microstructure refinement of the cement paste and reduces its porosity. In this the addition of glass powder contributes to the durability aspects of concrete [21]. Mortar and concrete performance tests to verify ASR from glass replacements are currently ongoing, and the results are planned for subsequent publications.

A surplus in reactive silica content in the glass has made it a suspect for causing expansions due to alkali–silica reaction (ASRs) in concrete. Previous studies show that at 20% replacements there is reduced risk for ASR; in fact finely milled glass powder is shown to mitigate ASR as it promotes the binding of silica with cement gel produced from pozzolanic reactions [19].

### 1.2. Research Objectives

1. To compare two recycling scenarios for flat glass waste, recycling to produce new glass and recycling as a cement replacement in concrete, for the resulting climate reduction and circularity, where climate reduction is assessed by reduced GWP and circularity by the share of recycled material consumed in the new glass and concrete products respectively.
2. To design a technical innovation involving a milling method to convert glass waste to an alternative binder that replaces cement in concrete, ascertained by:
  - Particle grading of glass powder.
  - Compressive strength of concrete.
  - Environmental impact: climate reduction and substitution of virgin material.
3. Assessing environmental impacts of recycling postconsumer cullet sourced from window renovations and demolition waste.
4. Analyzing climate reduction and circularity of glass powder as 20% cement replacement in concrete.
5. Analyzing climate and circularity gains in incorporating up to 33% glass in concrete as partial cement and aggregate substitution.

### 1.3. Short Description of Methodology

The environmental impact analysis is conducted by a consequential LCA using two premade LCAs available in the form of Environmental Product Declaration (EPD) for a commercial ready mixed concrete recipe and a new flat glass product. Each product describes the open and closed-loop recycling pathways respectively. Both recycling pathways are currently operational and are based on a Swedish case study conducted by the concrete research group at the University of Borås in Sweden [22]. The glass waste investigated in this article is sourced from windows during building renovation and demolition. The waste glass undergoes sorting and crushing at a recycling facility before the commencement of both recycling scenarios.

A consequential LCA is performed to determine the avoided environmental burden when glass is recycled as a cement replacement or used as a raw material in the production of new glass. The environmental impact of waste glass is calculated by weight percentage replacements in both recycling pathways.

The avoided burden by recycling glass waste in new glass production is calculated using a reference glass product, PLANICLEAR, with 14% recycled glass [23] in comparison with ORAE low-carbon glass with 65% recycled glass content [24].

The avoided burden by recycling glass waste in cement production is calculated using a reference concrete mix with 100% cement compared to mixes with 20% and 33% weight replacement of glass powder.

The climate impact reduction is assessed by GWP. To capture the benefits of replacing virgin material with waste, resource-based indicators such as abiotic depletion potential are chosen. Impacts from transport and recycling techniques form a minor part of the analysis.

The circularity of both recycling scenarios is calculated by the share of recycled content in the new glass and concrete product respectively. The recycled content is an indicator that accounts for the share of pre- and postconsumer input in the total product mass according to ISO 14021:2016 [25]. Building certification systems such as BREEAM and Miljöbyggnad in Sweden rely on the recycled content published in EPDs.

#### 1.3.1. Case—Closed-Loop Recycling

Following sorting and crushing operations, the glass waste is transported to Germany to produce new flat glass as the flat glass production facilities in Sweden are no longer operational. The analyzed glass product, ORAE low-carbon glass [24], contains 65% recycled glass as a substitute of virgin raw materials especially sand. Glass waste from production cut-offs or preconsumer cullet accounts for the major replacement share, ca 62–64%. Glass waste from demolition, i.e., postconsumer cullet, has a meagre share of 1–3% [23]. Therefore, the postconsumer cullet contributes only to a share of the avoided burden achieved by the total glass replacement in new glass production. The shared contribution of demolition waste to closed-loop recycling is determined by the mass allocation principle calculated by an allocation factor, α_postc,c_. The calculation of the allocation factor is demonstrated in the Materials and Methods Section.

#### 1.3.2. Case—Open-Loop Recycling

The technical innovation of glass milling has been developed by the research group. The feasibility of milled flat glass as a cement replacement is verified by binder properties such as particle grading and oxide composition, and concrete compressive strength at 28 days. The effects of glass as cement replacement are investigated in a concrete mix for 20% cement replacement by weight, the optimum replacement level recommended by previous research [19]. An additional concrete mix with glass powder as cement and as a fine aggregate replacement is investigated. In addition to the avoided burden of cement, this addresses the avoided burden of natural sand, especially the abiotic depletion potential arising from the extraction of natural sand.

There is a constant supply of waste glass from demolition the quantities of the flow are however variable, as observed in the case study preceding this article. The 20% replacement suits a low material flow scenario while the 33% replacement suits a larger material flow. The inclusion of larger amounts of glass waste in concrete gives the possibility for an increase in the circularity along with small winnings in climate impact reduction for the concrete product.

### 1.4. Contribution and Innovative Aspects

1. Along with the climate impact reduction, the recycling scenarios are evaluated on the basis of their circularity contributions.
2. Technical innovation showing a milling method to convert glass waste into a cement replacement in concrete. Evaluation of the cement replacement by particle grading and compressive strength of concrete.
3. A consequential LCA method with the possibility to integrate different glass and concrete products with established environmental profiles or EPDs.

## 2. Materials and Methods

### 2.1. Concrete Mix with Glass Replacement

An industrial concrete recipe of strength class C30/37 with a water–cement ratio of 0.5 was investigated. The experimental scheme consisted of three concrete mixes, with a reference with 100% cement, 20% glass as cement replacement by weight, 33% glass as 20% cement and 11% aggregate replacement respectively. The casting volume for each mix was 7 L, and the replacement of cement and aggregates with glass was performed as a direct replacement by weight. Since the mix volume was small, the volumetric changes due to weight-based replacement were not considered.

**Figure A1:**
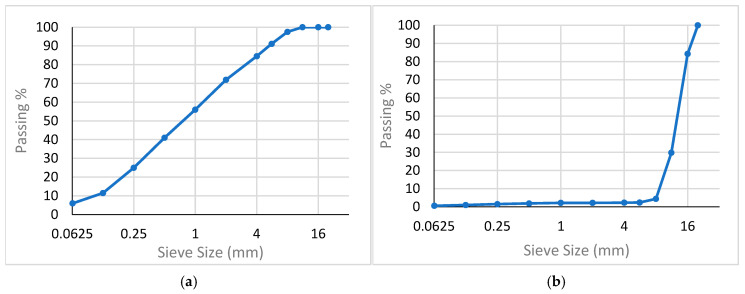
Grading curve for aggregate fractions (**a**) 8/16 mm and (**b**) 0/8 mm.

The fine and coarse aggregates were 0/8 mm natural sand and 8/16 mm crushed rock respectively. The grading curves and physical properties of the aggregate fractions are mentioned in Figure A1a,b and Table A1 respectively in Appendix A. The recipes for the concrete mixes are shown in Table 2. The oxide content of the glass powder is shown in Table 1, the particle size distribution which is a result of the milling process is shown in the results section.

The workability of the mix was determined by the slump test directly after casting according to SS-EN 12350-2:2019 [26]. The compressive strength was investigated at 28 days on cylinders of size 200 mm × 100 mm according to SS-EN 12390-3 [27].

### 2.2. Life Cycle Assessment

Life Cycle Assessment (LCA) was the method applied for the assessment of the environmental impact of the two recycling pathways in accordance with ISO 14044:2006 [28]. For a midpoint characterization, SS-EN 15804+A2:2019 [29] was followed. The LCA modelling was performed using Sima Pro version 10.2.0.3.

**Figure 3:**
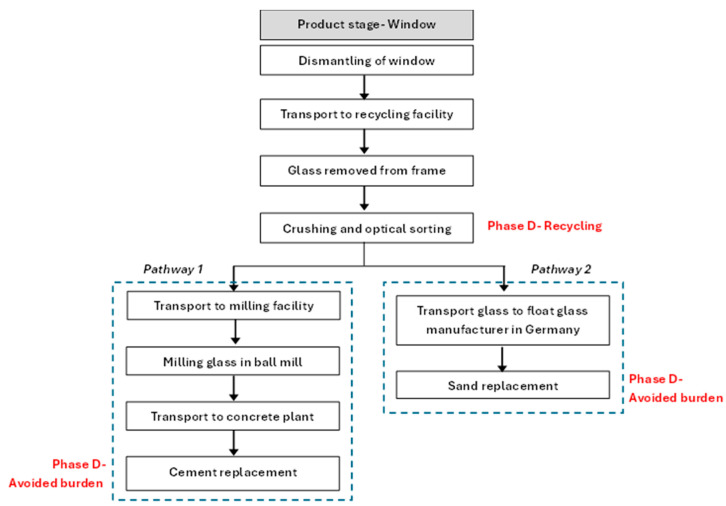
System boundary of LCA and recycling pathways, functional unit: 1 ton of glass waste from demolition.

The LCA was performed on an extended system boundary of the window glass as shown in Figure 3. The scope of the LCA was cradle to gate. The avoided burden approach was used to evaluate the recycling of glass as cement replacement and glass to produce new glass, referred to as pathways 1 and 2 respectively in Figure 3. Both pathways took a weight percentage replacement. As both glass and cement are major emitters, the governing environmental impact was CO_2_ emissions, analyzed by the GWP indicator. To capture the benefits of replacing virgin material with waste, resource-based indicators such as abiotic depletion potential were chosen.

The avoided burden method assumes that recycled glass substitutes an equivalent amount of virgin material in the reference system. A limitation is that the results depend on assumptions regarding substitution ratios and the environmental profile of the substituted material.

The glass removal, crushing and sorting processes took place in the recycling facility and were driven by electricity; the process flow is shown in Figure 3. The crushing and sorting process is crucial both for producing glass powder as cement replacement and cullet for new glass production. Since the processes are common for both recycling pathways, they were excluded from the system boundary for the assessment of environmental impacts.

An approximation of the energy consumed by the crushing and sorting processes was conducted in a preliminary study connected to this article to 7.5 kWh per ton of flat glass waste [22]. This is much lower than 12 kWh per ton, the energy consumption for the electricity-driven crushing and sorting process, as shown in previous research from China [7]. The differences in energy consumption values could be due to differences in the type of equipment and logistical support at the respective recycling facilities in Sweden and China.

### 2.3. Life Cycle Inventory—Recycling Glass as a Cement Replacement

This study assumes a hypothetical scenario for industrial-scale recycling with a value chain composed of a real-time glass recycler, milling facility and concrete producer. Their locations are Swedish cities of Örebro, Trollhättan and Borås, respectively.

#### 2.3.1. Activation of Glass by Milling

**Figure 4:**
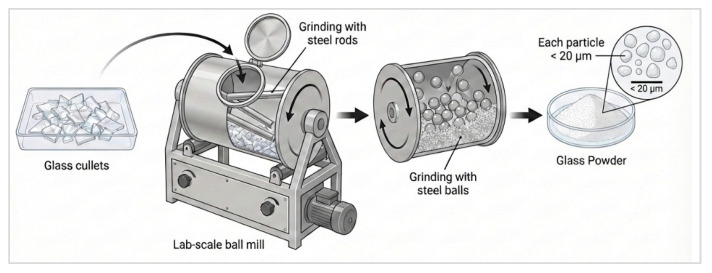
Recycling glass waste to cement replacement, activation by milling process [20].

Technically glass reacts as a pozzolan when milled to cement particle size, which is 20 µm. Glass as cement replacement is still to be implemented on an industrial scale in Sweden, with the milling process investigated solely on a laboratory scale. The milling process was carried out at the University of Borås in Sweden, and the milling routine is shown in Figure 4 [30].

Cullet of size 30–60 mm weighing 800 g was milled in a Nordic ball mill with an internal diameter of 206.5 mm, an internal length of 335 mm, and a thickness of 6 mm. The rotation speed of the ball mill was 90 ± 3 revolutions per minute for a total duration of 90 min. The milling process commenced with steel rods as milling media for 30 min followed by milling with steel balls 11, 15 mm in diameter of 7 kg each for the rest of the duration.

From a practical standpoint, the industrial implementation of glass powder as cement replacement requires an industrial-scale ball mill. A scaling up of the milling process from laboratory to industrial scale is attempted here.

The electricity demand of the laboratory-scale ball mill for a single batch of milling was measured at 0.3 kWh. For an industrial-scale ball mill, energy consumption was scaled up according to Bond’s comminution theory where energy consumed relates to material hardness and particle size reduction from feed to product [31]. Bond’s equation for specific energy, E in [kWh/ton], is shown in Equation (1).

(1) $$E=10*W_{i}\left[\frac{1}{\sqrt{P_{80}}}-\frac{1}{\sqrt{F_{80}}}\right]$$

For W_i_, work index of glass =13.5 kWh/ton [23]; P_80_, product size at 80% passing = 26 µm (Figure 4); and F_80_, feed size at 80% passing = 50 mm or 50,000 µm gives E = 27 kWh/ton.

For calculating the environmental impact, the electricity assumed was the Swedish electricity mix from 2021, medium voltage Cutoff-U.

#### 2.3.2. Replacing Cement—Avoided Burden

The major impact of cement is the CO_2_ emissions from the calcination process during cement manufacture. Other impacts include abiotic depletion by the extraction of raw materials such as limestone and fossil fuels needed in the manufacturing process. By replacing a part of the cement with milled waste glass, the burden of environmental impacts of cement can be partially avoided. Glass behaves as a binder when milled as opposed to the calcination process, which is more emission-intensive.

Equation (2) shows the calculation of avoided burden as the difference in cement amounts, Δm_cement_, for concrete mixes: reference mix with cement amount $m_{cement,ref}$ and recycled mix with 20% waste glass and cement amount, $m_{cement,glass\;20\%}$. The quantities of Δm_cement_, m_cement,ref_ and m_cement,glass 20%_ are shown in later in the article.

(2) $$∆m_{cement}=m_{cement,ref}-m_{cement,glass\;20\%}$$

The avoided burden Δm_cement_ is then used to calculate the savings in environmental impact based on climate change and resource use indicators from the cement EPD. This study analyzes CEM II Industricement from Skövde produced by Heidelberg materials [32].

#### 2.3.3. Replacing Cement and Sand—Avoided Burden

The reference concrete mix has natural sand as a fine aggregate, which brings with it an environmental burden of depletion of natural resources. The avoidance of this burden was investigated with a concrete mix where waste glass replaces 11% of the sand along with 20% of the cement in the concrete mix, shown by the glass 33% mix in Table 3.

The avoided burden is the difference in sand amounts between the reference mix, $m_{sand,ref}$ and 33% glass replacement $m_{sand,glass\;33\%}$, calculated by Equation (3); the quantities are given in Table 3.

(3) $$∆m_{sand}=m_{sand,ref}-m_{sand,glass\;33\%}$$

Glass replaces 74 kg of cement in 1 m^3^ of concrete mix, glass 20%; this is assumed to bring an avoided burden equivalent to 74 kg of cement. In addition to 74 kg of cement, glass replaces 102 kg of sand in 1 m^3^ of concrete mix, glass 33%, assumed to bring an avoided burden corresponding to 102 kg of sand added to 74 kg of cement. The avoided burden for the 33% mix is $∆m_{sand}+∆m_{cement}$; the impacts are calculated from indicators for sand and cement EPDs respectively.

The natural sand used in the mix was from Örsås in Sweden. The EPD for the Swedish sand was not available; therefore, the indicators for environmental impact are taken from Norwegian natural sand [33], details in Table 4.

### 2.4. Life Cycle Inventory—Recycling Glass Waste to New Glass

The recycling of glass waste as raw material for new glass production is currently in practice in Sweden; sorting and crushing of window glass takes place at a recycling facility at Örebro. The product is a postconsumer cullet which is transported to Torgau in Germany to produce new glass by the float process. Details regarding the transportation of waste glass from the Örebro recycling facility in Sweden to the float glass producer in Torgau, Germany, are given in Table 5.

Background data and unit processes are selected from the Ecoinvent database version 3.x, using the system model “allocation, cut-off by classification”.

Transport unit processes

- Truck: market for transport, freight, truck > 32 metric ton, diesel, EURO6 (Cut-off, U).
- Boat: market for transport, freight, sea, ferry, heavy fuel oil (Cut-off, U).

The ferry dataset represents average sea ferry freight transport and may include Ro-Ro ferry transport.

#### Replacing Sand—Avoided Burden

The waste glass replaces 65% of sand in ORAE low-carbon glass [24]. According to the EPD, 62–64% comes from preconsumer cullet and only 1–3% is postconsumer cullet or sourced from demolition. The weight of 1 m^2^ ORAE glass is 10 kg.

To determine the environmental impacts solely from demolition waste, a mass allocation was carried out by calculating the allocation factor, α_postc,c_. Simply put, the allocation factor is a ratio of the mass of postconsumer cullet to the mass of total cullet used in new glass production. The allocation factor for the reference glass PLANICLEAR and recycled glass ORAE is calculated in Table 6.

It is due to this allocation factor that the avoided burden was calculated. The avoided burden was calculated as the difference in postconsumer cullet content, Δm_postc,c_ between a reference flat glass product m_postc,planiclear_ and the recycled product, m_postc,orae_ according to Equation (4). The postconsumer cullet content is shown in Table 6.

(4) $$∆m_{postc,c}=m_{postc,planiclear}-m_{postc,orae}$$

The reference product is PLANICLEAR glass with postconsumer cullet share of 1% and total cullet share of 14% [23]. Both glass products are produced in the Torgau facility owned by Saint Gobain in Germany and have the same material properties.

Impact indicators were taken from EPDs of ORAE and PLANICLEAR following SS-EN 15804+A2:2019 [29]. Declared unit (DU) of both EPDs was 1 m^2^ flat glass with a thickness of 4 mm. Glass mass per declared unit för both ORAÉ and PLANICLEAR was 10 kg per m^2^ flat glass.

There was a 51% difference in the recycled glass content between the reference glass PLANICLEAR and recycled glass ORAE. This resulted in an avoided burden of −5.1 kg/m^2^; of this, the postconsumer cullet or demolition glass waste contributed only −0.2 kg/m^2^ which is 3.92% of the total waste glass content. The preconsumer cullet sourced from the production facility had a share of 96.08% of the total cullet content.

Background data and unit processes are selected from the Ecoinvent database version 3.x, using the system model “allocation, cut-off by classification.”

For transport, truck: market for transport, freight, lorry > 32 metric ton, diesel, EURO 6 (Cut-off, U); boat: market for transport, freight, sea, ferry, heavy fuel oil (Cut-off, U). The ferry dataset represents average sea ferry freight transport and may include Ro-Ro ferry transport.

### 2.5. Life Cycle Impact Assessment

The impact assessment of the recycling of waste glass as cement replacement and waste glass for new glass production was calculated by the avoided burden method. This is the environmental savings credited to a system when recycled glass replaces virgin raw materials in new glass and cement in concrete, respectively.

The impact assessment was carried out using SS-EN 15804+A2:2019 [29] Method V1.00/EF 3.0 normalization and weighting set. The recycling pathways were assessed based on environmental impact, including climate impact and resource use, especially abiotic depletion. The chosen impact categories along with indicators, models and relevant units are listed in Table 7.

Impact categories are based on inventory for the global average, excluding Switzerland, according to Ecoinvent 3—allocation, cut-off by classification—unit.

#### 2.5.1. LCIA—Recycling Glass as Cement Replacement

The recycling of glass as cement and sand replacement in the 20% and 33% concrete mixes results in an avoided burden calculated using $∆m_{cement}$ and $∆m_{sand}+∆m_{cement}$ taking the values −74 kg and −102 kg + −74 kg respectively. The minus sign indicates the removal of cement, sand and the resulting reduction in environmental impacts. An example calculation for GWP, the global warming potential from greenhouse gas emissions, is demonstrated and resolved in Equation 5 for the concrete mix with 20% glass as cement replacement. The GWP is calculated for the functional unit of 1 ton of glass waste.

(5) $$GWP_{20\%\;glass\;mix}=\underset{Avoided\;burden\;cement/ton\;glass}{\underbrace{-\left[\frac{{GWP}_{cement}\times {∆m}_{cement}}{{∆m}_{cement}}\right]-\left[{GWP}_{transportofcement}\right]\;}}\underset{Added\;burden\;glass/ton\;glass}{\underbrace{+\left[{GWP}_{milling}\right]+\left[{GWP}_{transportofglass}\right]}}$$

$${GWP}_{20\%glassmix}=-\left[\frac{779\times 74}{74}\right]-\left[0.911\right]+\left[0.926\right]+\left[2.55\right]=-776\;{\mathrm{kgCO}}_{2}\mathrm{eq}/\mathrm{ton}\;\mathrm{glass}$$

The avoided burden of cement just from manufacturing, A1–A3, is dominating the GWP result. The additional environmental impact or added burden of glass comes from milling and transport, which is roughly 0.7–0.8% of the emissions from cement.

The sources for the GWP values for cement, diesel for road transport, and electricity for milling are given in Table 5.

The GWP results for 33% glass concrete mix along with other impact indicators can be seen in Section 3.3.

#### 2.5.2. LCIA—Recycling Glass for New Glass Production

The EPDs report indicators such as GWP for the total cullet content; this makes it difficult to discern GWP savings from cullet derived from demolition waste alone. This study has used an allocation factor, α_postc,c_, of 3.92% to determine indicators, particularly from postconsumer cullet arising from demolition glass waste. An example calculation for GWP, the global warming potential from greenhouse gas emissions, is demonstrated and resolved in Equation (6). The GWP is calculated for the functional unit of 1 ton of glass waste.

(6) $$GWP_{new\;glass,\;postc,c}=\underset{Avoided\;burden\;sand/ton}{\underbrace{-\left[\frac{{(GWP}_{Planiclear}-{GWP}_{Orae)}\times \alpha_{postc,c}}{{∆m}_{cullet}}\right]\;}}\underset{Added\;burden\;glass/ton}{\underbrace{+\left[{GWP}_{transport\;of\;glass}\right]}}$$

$${GWP}_{new\;glass,post,c,c}=-\left[\frac{\left(10.5-5.48\right)\times 3.92\%}{5.1\times 10^{-3}}\right]+\left[120\right]=63.4\;{\mathrm{kgCO}}_{2}\mathrm{eq}/\mathrm{ton}\;\mathrm{glass}$$

The share of demolition glass or postconsumer cullet, 3.92%, is so small that the added burden of transport exceeds the avoided burden. This results in a gain of CO_2_ emissions due to transport. The minimum share of postconsumer cullet, $\alpha_{postc,c}$, to even out the transport emissions is 12,2%. The calculation is shown in Equation (7).

(7) $$\left[\frac{\left(10.5-5.48\right)\times \alpha_{postc,c}}{5.1\times 10^{-3}}\right]=120;\alpha_{postc,c}=12.2\%$$

The GWP for the preconsumer cullet with allocation factor 96.08% is shown in Equation (8); this excludes the added burden of transport as the cullet is available within the production facility.

(8) $${GWP}_{new\;glass,prec,c}=-\left[\frac{{(GWP}_{Planiclear}-{GWP}_{Orae)}\times \alpha_{prec,c}}{{∆m}_{cullet}}\right]$$

$${GWP}_{new\;glass,prec,c}=-\left[\frac{\left(10.5-5.48\right)\times 96.08\%}{5.1\times 10^{-4}}\times 10^{-3}\right]=-945\;{\mathrm{kgCO}}_{2}\mathrm{eq}/\mathrm{ton}\;\mathrm{glass}$$

$${GWP}_{new\;glass,total\;\mathrm{cullet}}=63.4-945=-881.5\;{\mathrm{kgCO}}_{2}\mathrm{eq}/\mathrm{ton}\;\mathrm{glass}$$

The total GWP savings with the total cullet content of preconsumer and postconsumer cullet is −881.5 ton CO_2_eq/ton glass including the burden of transport. The GWP indicators represent the manufacturing of float glass, phases A1–A3. The sources for the GWP values for diesel for transport by road and sea are given in Table 7. The results for other impact indicators are shown in Section 3.3.

### 2.6. Recycled Content

In this article, recycling content is calculated specifically for the postconsumer input, i.e., glass waste from demolition in concrete mix and new glass products. Recycled content is calculated according to Equation 9. The results of recycled content for concrete mixes and new glass products are shown in Section 3.

(9) $$Recycled\;content=\frac{amount\;of\;demolition\;glass\;waste}{amount\;of\;raw\;material}$$

Figure 1, Figure 2 and Figure 4 in this study were created using the AI-assisted platform Figurelabs (Figurelabs, San Jose, CA, USA). Prompt-based figure generation was used to produce schematics aligned with the material systems investigated. All the AI-generated outputs were iteratively refined and manually edited by the authors to ensure scientific accuracy, visual clarity, and consistency with the described methods.

## 3. Results and Discussion

### 3.1. Milling Process

**Figure 5:**
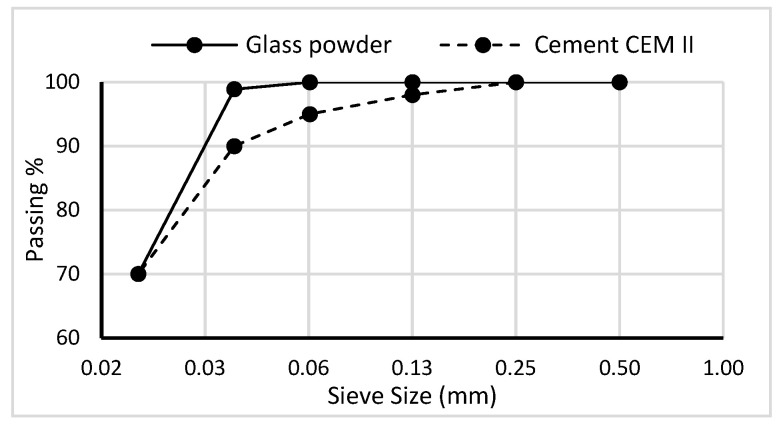
Particle size distribution of glass powder, cement CEM II, Industricement Skövde.

The milling produces glass powder with 70% of particles passing a 20 µm sieve; the particle size distribution of the glass powder and Swedish cement CEM II is shown in Figure 5. The glass powder fulfills the puzzolanic criteria of ASTM standard C1866M-20 with 95% of the particles passing the 45-micron sieve.

According to previous research, the energy consumed for milling cement clinker on an industrial scale is 32–37 kWh/ton [34]. The energy estimation from upscaling the glass milling process results in 27 kWh/ton which is slightly less than the energy consumed in milling cement. This can be attributed to the higher bond work index of cement, 14.95 kWh, in comparison to glass, 13.5 kWh/ton [35].

### 3.2. Compressive Strength of Concrete Mixes

The mean compressive strength of 3 concrete cylinders is shown in Table 8 for the concrete mixes with glass, along with the workability of the mix. The glass mixes show slightly higher workability than the reference despite having the same superplasticizer amount.

The compressive strength of concrete with 20% glass replacement is about 10% lesser than the reference concrete. Similar results have been seen in previous research for concrete with a water–cement ratio of 0.48 where the 28-day compressive strength for 20% glass and reference mixes are 37 MPa and 43 MPa respectively [36].

The compressive strength of the concrete mix 33% glass matches the reference concrete strength, with glass powder replacing 20% of the cement and 13% of the sand. This could be attributed to the effectivity factor of the glass, called k-value, which is the amount of SCM required to produce the same compressive strength as a given mass of Portland cement. As glass powder shows lower compressive strength at 20% replacement, it is sure to take a k-value less than cement, k < 1. This means that there needs to be more glass than 20% for the binder to show pozzolanic properties corresponding to 20% of the cement amount. There are many investigations ongoing to determine the k-value of glass; k is calculated to 0.4 for percentage replacements less than 50%, according to a previous study [37].

A scientific publication is underway on the k-value investigations into the glass powder investigated in this article. The experimental scheme also includes tests for durability, especially tests for alkali–silica reaction.

### 3.3. Results of Life Cycle Impact Assessment

The environmental impacts of the concrete mixes for 20% and 33% replacements are shown in Table 9. The avoided burden approach was used to calculate the avoided carbon dioxide emissions and abiotic depletion with the inclusion of glass in new glass and concrete respectively. This includes the impact of the avoided burden of cement along with the impact of transport. There is an increase in the CO_2_ emission savings with an increase in glass powder replacement, from 776 kg/ton for 20% to 792 kg/ton for 33%. A similar increase is also observable among the resource-based indicators ADP—minerals and fossil fuels.

The impacts of closed-loop recycling are categorized as avoided burden of sand, transport and total as shown in Table 10. The transport distances correspond to the Swedish case study, where glass sorting and crushing take place in Sweden. Following this the cullets are transported to Torgau in Germany to be used in new glass production as the flat glass production is not currently ongoing in Sweden. However, the transport burden is reduced by a higher margin in countries in Eastern Europe where there are more glass production facilities with shorter transportation distances between them.

### 3.4. Recycled Content

The recycled content for closed-loop recycling is shown by the share of postconsumer cullet or demolition glass waste to the total cullet content; the results are shown in Table 11. The recycled content for open-loop recycling is shown by the share of demolition glass waste to the total concrete constituents, in this case virgin raw materials; the results are shown in Table 12.

### 3.5. More Recycled Content, Lower Climate Impact

**Figure 6:**
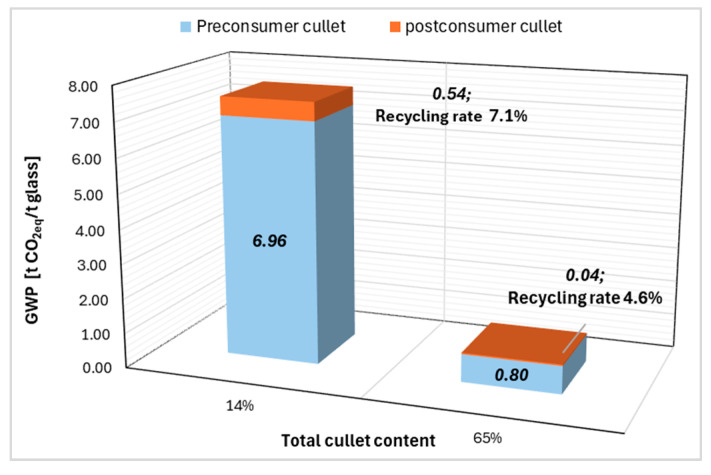
Recycled content and resulting climate impact, closed-loop recycling (excluding transport).

The GWP per ton glass cullet is calculated for Orae and Planiclear glass from their respective EPDs; the products show a recycled content of 14% and 65% respectively, as shown in Figure 6.

The preconsumer cullet forms a major share, 93% and 95% of the total cullet content for planiclear and orae glass respectively. Share of postconsumer glass is a meagre 7% and 4% of the total cullet content due to stringent manufacturing and product requirements of the flat glass, shown by orange columns in Figure 6.

Closed-loop recycling of waste glass is showing a significant reduction in climate impact with an increase in the recycled content. By increasing the recycling rate from 14% to 65%, the GWP shows a considerable reduction. This shows that the use of waste glass as a raw material in the production of new glass brings a significant reduction in CO_2_ emissions. Therefore, the closed-loop recycling pathway brings large climate impact reductions and circularity.

The GWP for the concrete mixes is calculated for cement and sand from their respective EPD’s and is plotted across glass replacement percentages. The coarse aggregates, water and superplasticizer contents are not counted as the quantities are the same for all mixes.

**Figure 7:**
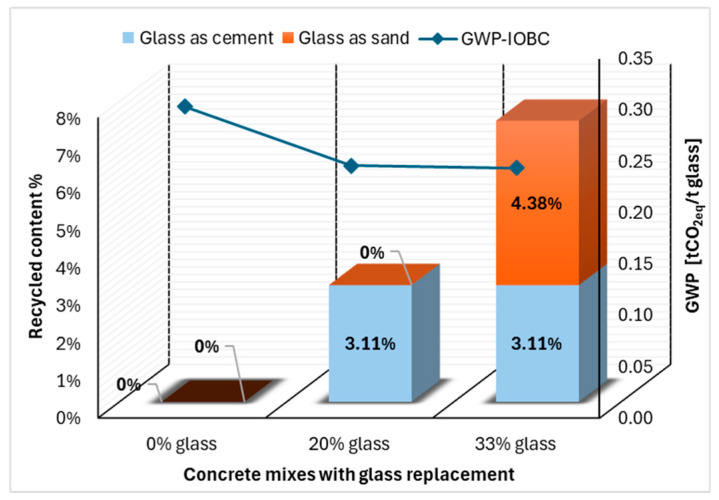
Recycled content and resulting climate impact, open-loop recycling (excluding transport and milling).

By recycling demolition glass as a cement replacement at 20%, a corresponding climate impact reduction is shown in comparison with reference concrete with 0% glass replacement. The climate reduction is 20%, from 0.3 t CO_2_eq to 0.25 t CO_2_eq per ton of demolition glass waste. For a 1 m^3^ concrete mix this corresponds to a recycled content of 3.11%, Figure 7.

The concrete mix 33% glass shows a very slight decrease in climate reduction compared to 20% glass mix despite the increased recycled glass content. The sand in the concrete mix has a much lower carbon footprint compared to cement. Therefore, even when the recycled content is increased, i.e., more substitution of glass powder, the CO_2_ savings are very minimal.

The replacement of glass powder as cement and sand replacement is advantageous from a waste minimization perspective. This gives the possibility to replace more glass in the concrete and in the process gain more compressive strength. It is, however, important to validate the concrete mix for durability aspects such as ASR, which can reduce the service life of the concrete. The need for repair and reconstruction can influence the environmental profile of the concrete element, thereby affecting the lifecycle outcomes.

## 4. Conclusions

1. Waste glass recycled in new glass production shows larger climate impact reduction and circularity compared to recycling glass as a cement replacement in concrete. The CO_2_ emission savings for new glass production is −945 kgCO_2_eq/ton glass. When milled glass replaces 20% cement in the concrete, the avoided emissions amount to −776 kgCO_2_eq/ton glass.
2. The milling method is successful in producing glass powder as a cement replacement, with a particle size distribution comparable to cement. The compressive strength of the concrete mix with 20% glass is 38.7 MPa compared to the reference concrete with 43.8 MPa.
3. When glass powder partially replaces cement and sand in the concrete at 20% and 13% respectively, the compressive strength is 45 MPa, therefore fulfilling the reference concrete strength. This glass replacement of 33% is a good example for waste reduction, as it shows that it is possible to retain concrete performance while increasing the recycled content in the concrete mix. The durability of the concrete needs to be investigated.
4. To ensure the quality of flat glass, only 1–3% of demolition glass can be used in the production of new glass, the rest of the waste glass being sourced from production cut-offs. In this way the contribution of demolition glass waste to the climate impact reduction is only −38 kgCO_2_eq/ton glass, which the total recycled glass contributes to −945 kgCO_2_eq/ton glass. Alternatively, it is possible to replace 20, 33% demolition glass in concrete without losing compressive strength.
5. There may be uncertainty in the LCA results as the pre-recycling process, phase D, has not been taken into account.

## Figures and Tables

**Table 1:** Oxide composition of flat glass, OPC [19].

Oxides	Flat Glass	OPC
SiO_2_	71.2	20.6
Al_2_O_3_	0.36	5.6
Fe_2_O_3_	0.44	3.0
CaO	9.33	62.4
MgO	3.86	3.0
Na_2_O	13.22	1–2
K_2_O	0.04	1–2

**Table 2:** Recipe for concrete mixes for 1 m^3^, C30/37, w/c 0.5.

Mix Constituents	Glass 0% [kg/m^3^]	Glass 20% [kg/m^3^]	Glass 33% [kg/m^3^]
Cement-CEM II/A-LL 52.5 R	370	296	296
Glass powder	0	74	176
Natural sand 0/8 mm	1004	1001	870
Crushed rock 8/16 mm	806	804	806
Water	192	192	192
Superplasticizer Sika Viscocrete 6720	3.7	3.7	3.7

**Table 3:** Avoided burden cement, sand for 1 m^3^ of concrete mix.

Mix Constituents	Reference [kg/m^3^]	Glass 20% [kg/m^3^]	$∆m_{cement}$ [kg/m^3^]	Glass 33% [kg/m^3^]	$∆m_{sand}$ [kg/m^3^]
Cement	370	296	−74	296	
Glass powder	0	74		176	
Natural sand 0/8 mm	1004	1001		870	−102

**Table 4:** Inventory for LCA calculation.

Parameter	Source	Details
Electricity mix	Ecoinvent 3	Electricity 2021, medium voltage (SE), Cutoff-U Declared unit 1 kWh
Road transport		Lorry Euro 6, >32 tons, diesel
Sea transport		RoRo ferry, diesel
Cement- CEM II/A-LL 52.5R	EPD	Industricement, Skövde Heidelberg [32], DU 1ton
Natural sand		ADDA Tørket sand (dry sand), size: 0–8 mm [33], DU 1ton
Orae glass		Orae 4 mm low-carbon glass, Saint Gobain [24], DU 1 m^2^
Planiclear glass		Planiclear 4 mm Clear float glass, Saint Gobain [23], DU 1 m^2^

**Table 5:** Transport associated with closed and open-loop recycling of glass, Swedish case study.

Transport Locations	Mode of Transport	Fuel Type	Distance [km]
Glass waste as cement replacement			
Örebro-Trollhättan	Road-truck	Diesel	121
Trollhättan-Borås			97
Glass waste to new glass production			
Örebro-Gedser	Road-truck	Diesel	689
Gedser-Rostock	Sea-RoRo Ferry		49
Rostock-Torgau	Road-truck		353

**Table 6:** Avoided burden sand for recycling of glass to produce new glass.

	PLANICLEAR Reference	ORAE	$∆m_{postc,c}$
Waste glass content [weight %]	14	65	
m_cullet_ [kg/m^2^]	0.14 × 10 = 1.4	0.65 × 10 = 6.5	6.5 − 1.4 = 5.1
m_postc,c_ [kg/m^2^]	1% 0.01 × 10 = 0.1	3% 0.03 × 10 = 0.3	0.3 − 0.1 = 0.2
$\mathrm{Allocation}\;\mathrm{factor}\;\mathrm{for}\;\mathrm{postconsumer}\;\mathrm{cullet},\;\alpha_{\mathrm{postc},c}=\frac{m_{postc,c}}{m_{cullet}}$			$\frac{0.2}{5.1}=3.92\%$
$\mathrm{Allocation}\;\mathrm{factor}\;\mathrm{for}\;\mathrm{preconsumer}\;\mathrm{cullet},\;\alpha_{\mathrm{prec},c}=100\%-\frac{m_{postc,c}}{m_{cullet}}$			96.08%

**Table 7:** Life Cycle Impact categories and indicators.

Category	Indicator	Unit	Model/Method
Climate impact	Global warming potential excluding biogenic carbon GWP-IOBC	kg CO_2_eq	IPCC 2013
Abiotic depletion	ADP—minerals and metals	kg Sb eq	CML 2001
	ADP—fossil fuels	MJ	

**Table 8:** Compressive strength of concrete mixes with glass powder.

	Replacements in Weight %		
	Glass 0%	Glass 20%	Glass 33%
Workability [mm]	180	185	195
Compressive strength			
Mean [MPa]	43.8	38.7	45
Standard deviation [MPa]	3.6	2	4.6

**Table 9:** LCIA indicators for concrete mixes with 20%, 33% per ton replacement of glass waste.

Concrete Mix	GWP-IOBC [kg CO_2_eq/ton]	ADP—Minerals and Metals [ton Sb eq/ton]	ADP—Fossil Fuels [MJ/ton]
20% glass	−776	−2.88 × 10^−4^	−3499.65
33% glass	−792	−2.96 × 10^−4^	−3581.35
	2.06%	2.77%	2.33%

**Table 10:** LCIA indicators for closed-loop recycling allocated for postconsumer glass waste.

Phases [/ton glass]	GWP-IOBC [kg CO_2_eq/ton]	ADP—Minerals and Metals [kg Sb eq/ton]	ADP—Fossil Fuels [MJ/ton]
Avoided burden of sand allocated to postconsumer cullet	−38.5	4.14 × 10^−5^	−514.2
Transport	120	3.25 × 10^−4^	204.99
Avoided burden including transport	82.01	3.27 × 10^−4^	−309.2

**Table 11:** Recycled content in new glass shown by weight % of postconsumer cullet.

	Planiclear 14% Cullet	Orae 65% Cullet
Total cullet (kg/m^2^)	1.40	6.50
Postconsumer cullet (kg/m^2^)	0.10	0.30
**Recycled content**	7.1%	4.6%

**Table 12:** Recycled content in concrete mixes containing shown by weight % glass powder.

Mix Constituents [kg/m^3^]	Glass 0%	Glass 20%	Glass 33%
Cement- CEM II/A-LL 52.5 R	370	296	296
Glass powder	0	74	176
Natural sand 0/8 mm	1004	1001	870
Crushed rock 8/16 mm	806	804	806
Water	182	182	182
Superplasticizer	3.7	3.7	3.7
**Recycled content**	0	3.11%	7.49%

## Data Availability

The original contributions presented in this study are included in the article. Further inquiries can be directed to the corresponding author.

## Abbreviations

The following abbreviations are used in this manuscript:

SCM	Supplementary Cementitious Material
EPD	Environmental Product Declaration
OPC	Ordinary Portland Cement
LCA	Life Cycle Assessment
GWP	Global Warming Potential
GWP-IOBC	Global Warming Potential—Instant Oxidation of Biogenic Carbon
ADP	Abiotic Depletion Potential

## Appendix A. Aggregate Properties

**Table A1:** Properties of fine and coarse aggregates.

Physical Property	Standard	8/16 mm Crushed Stone	0/8 mm Natural Sand
Water Absorption 24 h [%]	SS-EN 1097-6:2013 [38]	0.5	0.3
Apparent Density [g/cm^3^]	SS-EN 1097-6:2013 [38]	2.64	2.65
Flakiness index [%]	SS-EN 933-3:2012 [39]	11.7	5.3
Loose Bulk Density [g/cm^3^]	ASTM C29/29M [40]	1.37	1.45

## References

[1] European Commission Buildings and Construction—Industry & Sustainability.

[2] Zink T., Geyer R. (2017). Circular Economy Rebound. J. Ind. Ecol..

[3] Fredriksson A., Janné M., Bäckstrand J., Razaq B., Sadagopan M., Harale N., Nagy A. (2025). Scaling Recycled Concrete in Sweden—Technical Advances, Supply Chains and Optimized Logistics for a Circular Flow. Nord. Concr. Res..

[4] (2020). Glass for Europe. Flat Glass in Climate-Neutral Europe. https://glassforeurope.com/wp-content/uploads/2020/01/flat-glass-climate-neutral-europe.pdf.

[5] Sonnentail C., Storm O., Berglund O.H., Löfås P., Colm T., Kordestani A., Bokström A., Sjösten O. (2020). Increased Circular Use of Flat Glass. https://databas.resource-sip.se/storage/Slutrapport%2046116-1.pdf.pdf#page=1.00.

[6] Boverket (2004). The Planning and Building Act and the Planning and Building Ordinance. https://www.boverket.se/en/start/laws-and-regulations/national-regulations/pbl-pbf/.

[7] Yuan X., Wang J., Song Q., Xu Z. (2024). Integrated assessment of economic benefits and environmental impact in waste glass closed-loop recycling for promoting glass circularity. J. Clean. Prod..

[8] Zero Waste Europe (2022). How Circular Is Glass? A Report on the Circularity of Single-Use Glass Packaging.

[9] Hammond G., Jones C. (2025). Inventory of Carbon and Energy Version 4.

[10] Deschamps J., Simon B., Tagnit-Hamou A., Ben Amor M. (2018). Is open-loop recycling the lowest preference in a circular economy? Answering through LCA of glass powder in concrete. J. Clean. Prod..

[11] Kucukvar M., Egilmez G., Tatari O. (2016). Life cycle assessment and optimization-based decision analysis of construction waste recycling for a LEED-certified university building. Sustainability.

[12] Scalet B.M., Garcia M.M., Sissa A.Q., Roudier S., Delgado S.L. (2013). Best Available Techniques (BAT) Reference Document for the Manufacture of Glass.

[13] Hestin M., de Veron S., Burgos S. (2016). Economic study on recycling of building glass in Europe. Deloitte Sustain.

[14] Glass for Europe (2024). Recycling of End-of-Life Building Glass—A Powerful Tool to Reduce CO_2_ Emissions. https://glassforeurope.com/wp-content/uploads/2025/05/GfE-Recycling-of-end-of-life-building-glass_2024.pdf.

[15] European Recycling Industries’ Confederation (EuRIC), Recycling Europe, FERVER, FEAD (2025). Joint Position Paper on Recycling. https://euric.org/images/Position-papers/Final_Joint_Position_Paper_-_Recycling_Europe_FERVER_FEAD_3.11.2025.pdf.

[16] Cheng D., Reiner D.M., Yang F., Cui C., Meng J., Shan Y., Liu Y., Tao S., Guan D. (2023). Projecting future carbon emissions from cement production in developing countries. Nat. Commun..

[17] CEMBUREAU (2024). From Ambition to Deployment—Our 2050 Roadmap.

[18] Santos-Ortega J.L., Ferreiro-Cabello J., Fraile-García E., Somovilla-Gómez F. (2025). Applying the Life Cycle Assessment to the Use of Biochar from Vine Pruning Waste as an Additive in Mortar. Materials.

[19] Al-Hellali N., Bengtsson M., Nagy A., Sadagopan M. (2025). Glass waste as a supplementary cementitious material in climate reduced concrete–A review. Nord. Concr. Res..

[20] (2020). Standard Specification for Ground-Glass Pozzolan for Use in Concrete.

[21] Kamali M., Ghahremaninezhad A. (2016). An investigation into the hydration and microstructure of cement pastes modified with glass powders. Constr. Build. Mater..

[22] Almasri G., Talostan S. (2025). Potential of Flat Glass Recycling in Sweden—An Experimental Study with LCA Analysis. Bacherlor’s Thesis.

[23] EPD International AB (2021). Planiclear 2–19 mm Clear Float Glass.

[24] EPD International AB (2023). Oraé Low Carbon Glass 4 mm.

[25] (2016). Environmental Labels and Declarations—Self-Declared Environmental Claims (Type II Environmental Labelling).

[26] (2019). Testing Fresh Concrete—Part 2: Slump Test.

[27] (2019). Testing Hardened Concrete—Part 3: Compressive Strength of Test Specimens.

[28] (2006). Environmental Management—Life Cycle Assessment—Requirements and Guidelines.

[29] (2019). Sustainability of Construction Works—Environmental Product Declarations—Core Rules for the Product Category of Construction Products.

[30] Sadagopan M., Loubani H., Kadawo A., Nagy A., Bashouri I., Bashouri M., Mnassir J. (2024). Optimized Milling Technique for Glass Recycling: Glass Waste as Cement Replacement.

[31] Hosten C., Avsar C. (1998). Grindability of mixtures of cement clinker and trass. Cem. Concr. Res..

[32] Heidelberg Materials Cement Sverige AB (2024). Industricement Skövde.

[33] Adda Byggkjemi AS (2024). ADDA Torket Sand (Dry Sand), Size: 0–8 mm.

[34] Rajczakowska M., Kothari A., Buasiri T., Cwirzen A. (2025). Recycled and mechanically activated concrete fines as a complete substitute for Portland cement: Feasibility and life cycle assessment. Case Stud. Constr. Mater..

[35] Tsakalakis K.G. Bond Work Index Tables (wi) from Various Sources.

[36] Islam G.M.S., Rahman M.H., Kazi N. (2017). Waste glass powder as partial replacement of cement for sustainable concrete practice. Int. J. Sustain. Built Environ..

[37] del Toro E.M.G., Alcala-Gonzalez D., Más-López M.I., García-Salgado S., Pindado S. (2021). Use of ecofriendly glass powder concrete in construction of wind farms. Appl. Sci..

[38] (2013). Tests for Mechanical and Physical Properties of Aggregates—Part 6: Determination of Particle Density and Water Absorption.

[39] (2012). Tests for Geometrical Properties of Aggregates—Part 3: Determination of Particle Shape—Flakiness Index.

[40] (2023). Standard Test Method for Bulk Density (“Unit Weight”) and Voids in Aggregate.

